# Frequency of Use Among Middle and High School Student Tobacco Product Users — United States, 2015–2017

**DOI:** 10.15585/mmwr.mm6749a1

**Published:** 2018-12-14

**Authors:** Gabriella M. Anic, Michael D. Sawdey, Ahmed Jamal, Katrina F. Trivers

**Affiliations:** ^1^Office of Science, Center for Tobacco Products, Food and Drug Administration, Beltsville, Maryland; ^2^Office on Smoking and Health, National Center for Chronic Disease Prevention and Health Promotion, CDC.

## Abstract

Tobacco product use during adolescence increases the risk for lifelong nicotine addiction and immediate adverse health effects (*1*,*2*). During 2011–2017, current use of cigarettes, cigars, smokeless tobacco, and pipe tobacco decreased significantly among middle and high school students, but current use of e-cigarettes increased significantly from 1.5% to 11.7% (*3*). In 2017, an estimated 19.6% of high school students (2.95 million) and 5.6% of middle school students (0.67 million) were current users of any tobacco product; e-cigarettes were the most commonly used tobacco product for both middle (3.3%) and high (11.7%) school students (*3*). The Food and Drug Administration (FDA) and CDC analyzed combined data from the 2015–2017 National Youth Tobacco Surveys (NYTS) to determine past 30-day (current) frequency of use of cigarettes, e-cigarettes, cigars, smokeless tobacco, and hookahs among U.S. high school and middle school students. During 2015–2017, the proportion of students currently using tobacco products who used a product for ≥20 of the past 30 days ranged from 14.0% of cigar smokers to 38.7% of smokeless tobacco users among high school students and from 13.1% of e-cigarette users to 24.5% of hookah smokers among middle school students. Among current users, use of two or more tobacco products ranged from 76.7% (e-cigarettes) to 90.9% (hookahs) among those using the product ≥20 of the preceding 30 days, from 68.0% (e-cigarettes) to 84.2% (hookahs) among those using the product for 6 to 19 of the preceding 30 days, and from 48.8% (e-cigarettes) to 77.2% (cigarettes) among those using the product for 1 to 5 of the preceding 30 days. Sustained implementation of proven tobacco control strategies focusing on all types of tobacco products, in coordination with the regulation of tobacco products by FDA, are needed to reduce tobacco product initiation and use among U.S. youths.

NYTS is a cross-sectional, school-based, pencil-and-paper survey administered to U.S. middle (grades 6–8) and high (grades 9–12) school students (*4*). A three-stage cluster sampling procedure was used to generate a nationally representative sample of U.S. students attending public and private schools in grades 6–12. Data were combined from the 2015 (17,711), 2016 (20,675), and 2017 (17,872) NYTS to provide a sufficient sample size to assess different categories of use frequency. Response rates for 2015–2017 were 63.4%, 71.6%, and 68.1%, respectively. Information on current use (≥1 day in the past 30 days) was collected for the following tobacco products: cigarettes, cigars (cigars, cigarillos, or little cigars), smokeless tobacco products (chewing tobacco, snuff, dip, snus, or dissolvable tobacco products), e-cigarettes, hookahs (water pipes used to smoke tobacco), pipe tobacco, and bidis (small imported cigarettes wrapped in a leaf). Information on frequency of use (number of days used in the past 30 days) was collected for five tobacco products: cigarettes, cigars, smokeless tobacco, e-cigarettes, and hookahs. Frequency of hookah smoking was collected only in 2016 and 2017; the other four products were assessed in 2015, 2016, and 2017. Frequency of use information was not collected in any of the surveys for pipe tobacco, bidis, and certain specific smokeless tobacco products (snus and dissolvable tobacco products). Response options describing self-reported frequency of use were “0 days,” “1–2 days,” “3–5 days,” “6–9 days,” “10–19 days,” “20–29 days,” and “all 30 days.” Frequent use was defined as using a product for ≥20 of the preceding 30 days. Multiple tobacco product use was defined as any past 30-day use of two or more tobacco products among current users of cigarettes, cigars, e-cigarettes, smokeless tobacco, and hookahs separately. Students with missing responses for frequency of use were excluded from the analysis.* Students missing data on current use of individual products were considered nonusers of that product. National prevalence estimates were calculated with 95% confidence intervals, and weighted population counts were rounded down to the nearest 10,000; all estimates were time-averaged over the pooled survey years. Survey weights were used to account for the complex survey design and adjusted for nonresponse.

During 2015–2017, among high school students who were current users of each product, the prevalence of frequent use (≥20 of the past 30 days) was as follows: 28.4% of cigarette smokers (330,000), 17.4% of e-cigarette users (330,000), 14.0% of cigar smokers (160,000), 38.7% of smokeless tobacco users (260,000), and 16.7% of hookah smokers (60,000) (Table). Among middle school students, the prevalence of frequent use was 17.5% of cigarette smokers (40,000), 13.1% of e-cigarette users (60,000), 13.2% of cigar smokers (20,000), 21.5% of smokeless tobacco users (30,000), and 24.5% of hookah smokers (20,000). High school student current users who used the product 1–5 of the past 30 days accounted for 50.3% of cigarette smokers, 61.4% of e-cigarette users, 70.8% of cigar smokers, 45.2% of smokeless tobacco users, and 66.3% of hookah smokers. The proportion of middle school current users who used the product 1–5 of the past 30 days was 67.4% of cigarette smokers, 68.4% of e-cigarette users, 71.2% of cigar smokers, 56.0% of smokeless tobacco users, and 61.8% of hookah users.

**TABLE:** Frequency of use (number of days of use during the preceding 30 days) among middle and high school students currently using cigarettes, e-cigarettes, cigars, smokeless tobacco, and hookahs* — National Youth Tobacco Survey, United States, 2015–2017

Days of use	Cigarettes		E-cigarettes		Cigars		Smokeless tobacco		Hookahs^†^	
	% (95% CI)	Estimated no. of users^§^	% (95% CI)	Estimated no. of users^§^	% (95% CI)	Estimated no. of users^§^	% (95% CI)	Estimated no. of users^§^	% (95% CI)	Estimated no. of users^§^
**High school**										
1–2	35.9 (33.2–38.6)	440,000	41.3 (38.8–43.9)	790,000	51.7 (49.1–54.2)	610,000	32.9 (29.5–36.5)	220,000	49.2 (45.0–53.4)	190,000
3–5	14.4 (12.8–16.3)	170,000	20.1 (18.2–22.0)	380,000	19.2 (17.4–21.2)	220,000	12.3 (10.3–14.5)	80,000	17.1 (14.2–20.5)	60,000
6–9	8.9 (7.6–10.3)	100,000	10.7 (9.4–12.1)	200,000	7.9 (6.7–9.3)	90,000	7.2 (5.7–9.1)	50,000	11.1 (8.6–14.1)	40,000
10–19	12.5 (10.9–14.2)	150,000	10.5 (9.4–11.9)	200,000	7.2 (6.1–8.6)	80,000	8.9 (7.2–11.0)	60,000	5.9 (4.4–7.8)	20,000
20–29	8.9 (7.5–10.6)	100,000	5.3 (4.5–6.4)	100,000	3.5 (2.8–4.4)	40,000	6.5 (5.0–8.4)	40,000	3.7 (2.6–5.2)	10,000
30	19.4 (17.1–22.0)	230,000	12.1 (10.6–13.7)	230,000	10.5 (9.0–12.3)	120,000	32.2 (27.8–37.0)	220,000	13.0 (10.1–16.6)	50,000
**Middle school**										
1–2	48.7 (43.0–54.3)	120,000	50.8 (47.2–54.5)	250,000	57.5 (51.2–63.6)	110,000	45.2 (38.6–51.9)	80,000	41.6 (33.7–49.9)	50,000
3–5	18.8 (14.4–24.2)	40,000	17.6 (15.0–20.5)	80,000	13.7 (10.2–18.0)	20,000	10.8 (8.0–14.3)	10,000	20.2 (15.0–26.6)	20,000
6–9	7.9 (5.4–11.5)	20,000	9.9 (8.2–12.0)	50,000	10.3 (7.0–14.8)	20,000	15.1 (10.6–21.0)	20,000	10.2 (6.9–14.7)	10,000
10–19	7.1 (5.0–9.8)	10,000	8.6 (6.7–10.9)	40,000	5.3 (3.4–8.2)	10,000	7.5 (4.2–13.0)	10,000	**—^¶^**	**—^¶^**
20–29	4.8 (2.9–7.7)	10,000	4.2 (3.0–5.9)	20,000	**—^¶^**	**—^¶^**	**—^¶^**	**—^¶^**	**—^¶^**	**—^¶^**
30	12.8 (10.0–16.3)	30,000	8.9 (7.1–11.1)	40,000	11.3 (8.2–15.4)	20,000	17.9 (13.1–23.9)	30,000	20.9 (15.4–27.9)	20,000

**Abbreviation:** CI = confidence interval.

* Frequency of current use of cigarettes, e-cigarettes, cigars (defined as cigars, cigarillos, or little cigars), smokeless tobacco (defined as chewing tobacco, snuff, or dip), and hookahs was determined by asking participants on how many days they used each of these tobacco products during the preceding 30 days.The percentages given indicate the proportion of users for each product (e.g., 35.9% of cigarette users use that product 1–2 days per month.)

^†^ Hookah estimates were based on data from 2016 and 2017. Frequency of hookah smoking was not asked in the 2015 survey.

^§^ Estimated number of users was rounded down to the nearest 10,000.

**^¶^** Data are statistically unreliable because the relative standard error was >30%.

Among middle and high school students who used any of these five products on ≥20 of the preceding 30 days, multiple tobacco products were used by 87.5% of cigarette smokers, 76.7% of e-cigarette users, 81.6% of cigar smokers, 77.0% of smokeless tobacco users, and 90.9% of hookah smokers (Figure). Similarly, for middle and high school students who currently used a product for 1–5 of the preceding 30 days, multiple tobacco product use was reported for 77.2% of cigarette smokers, 48.8% of e-cigarette users, 72.0% of cigar smokers, 72.4% of smokeless tobacco users, and 70.5% of hookah smokers.

**FIGURE:**
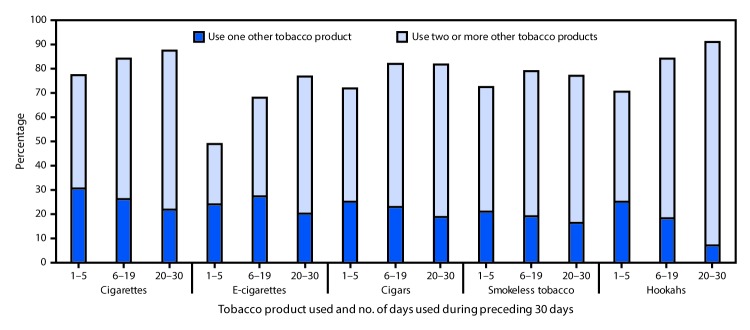
Percentage of middle and high school students who were current users of cigarettes, e-cigarettes, cigars, smokeless tobacco, and hookahs, who reported multiple tobacco product use,* by number of days used during the preceding 30 days — National Youth Tobacco Survey, United States, 2015–2017 * Multiple tobacco product use was defined as a current cigarette smoker, e-cigarette user, cigar smoker, smokeless tobacco user, or hookah smoker also using at least one of the following products in the past 30-days: cigarettes; cigars (cigars, cigarillos, or little cigars); smokeless tobacco (chewing tobacco, snuff, or dip); e-cigarettes; hookahs; tobacco pipes; snus; dissolvable tobacco (dissolvables); and bidis.

## Discussion

E-cigarettes were the most commonly used tobacco product by middle and high school students in 2017, followed by cigars and cigarettes (*3.*) Use of tobacco products in any form by youths is unsafe, including infrequent use (*1*,*2*). During 2015–2017, the frequency of tobacco product use among current middle and high school users varied by product type and school level. However, for all assessed products, most current users reported using each product for 1–5 of the past 30 days. The products most commonly used ≥20 of the past 30 days by high school students were smokeless tobacco (38.7%) and cigarettes (28.4%) and by middle school students were hookahs (24.5%) and smokesless tobacco (21.5%).

Any frequency of tobacco product use might lead to symptoms of nicotine dependence (*5*). Symptoms of dependence, including strong cravings (14%), irritability and restlessness when not using tobacco products (11%), strong desire to use the product (6%), and wanting to use the tobacco product within 30 minutes of awakening (1%) have been reported by U.S. adolescent tobacco product users who use a single tobacco product on 1–2 of the previous 30 days (*5*). A high prevalence of multiple tobacco product use was observed for all products, regardless of the number of days that a tobacco product was used. The prevalence of reporting symptoms of nicotine dependence is 2–3 times higher for multiple product users than that for single product users (*5*). Given that nicotine dependence is a major determinant of whether a person becomes a long-term user of tobacco products, reducing experimentation by youths and initiation of all forms of tobacco product use is important to preventing future dependency on, and more frequent use of, these products (*1*,*2*,*6*).

The findings in this report are subject to at least four limitations. First, the data are self-reported; thus, the findings are subject to potential reporting bias. Second, data were not collected on the frequency of using tobacco pipes, snus, dissolvables, bidis, or by type of cigar. Although this precludes reporting frequency of use for these specific products, it should not affect the reported estimates of frequency of use of cigarettes, cigars, e-cigarettes, smokeless tobacco, and hookahs. Third, data were averaged across several years, although there were no significant changes in frequency of use during 2015–2017 for most products.^†^ Finally, NYTS only recruited students from public and private schools; therefore, the findings might not be generalizable to youths who are being home-schooled, have dropped out of school, or are in detention centers.

Understanding tobacco product use patterns, including frequency of use and multiple tobacco product use, is important for sustaining implementation of proven tobacco control strategies and regulation of all types of tobacco products. In 2009, FDA was granted immediate authority to regulate cigarettes, cigarette tobacco, roll-your-own tobacco, and smokeless tobacco^§^; in 2016, FDA issued a final rule that extended its regulatory authority to all other tobacco products (*7*). Regulation of tobacco products, along with implementing proven tobacco control and prevention strategies, can reduce the initiation and use of tobacco products among youths. Strategies to reduce youth tobacco product use include increasing the price of tobacco products, implementing advertising and promotion restrictions and national public education media campaigns, and raising the minimum age of purchase for tobacco products to 21 years (*1*,*2*,*8*,*9*). Monitoring the frequency of using tobacco products, including the use of multiple products, is important for informing these strategies to prevent and reduce youth tobacco product use.

**Summary**

**What is already known about this topic?**

Most tobacco product use begins during adolescence or young adulthood, increasing the risk for lifelong nicotine addiction and adverse health effects.

**What is added by this report?**

During 2015–2017, the proportion of students currently using cigarettes, cigars, e-cigarettes, smokeless tobacco, or hookahs who used each product ≥20 of the past 30 days ranged from 14.0% of cigar smokers to 38.7% of smokeless tobacco users among high school students and from 13.1% of e-cigarette users to 24.5% of hookah smokers among middle school students.

**What are the implications for public health practice?**

Understanding tobacco product use patterns including frequency of use is important for sustained implementation of proven tobacco control strategies and the regulation of tobacco products.

## Footnotes

* The ranges of proportions of those with missing responses for frequency of use questions were 2.1%–2.5% for cigarettes, 1.5%–2.3% for e-cigarettes, 2.1%–3.1% for cigars, 1.9%–3.2% for smokeless tobacco products, and 2.7%–3.4% for hookahs.

† A chi-squared test found no significant differences across years for the prevalence of infrequent, moderate, and frequent use of e-cigarettes (p = 0.11); cigars (p = 0.44); smokeless tobacco products (p = 0.66); and hookahs (p = 0.80). The prevalence of frequent cigarette smoking decreased during 2015–2017 (p = 0.01).

§ Family Smoking Prevention and Tobacco Control Act, Pub. L. 111–31, 123 Stat. 1776 (June 22, 2009). https://www.gpo.gov/fdsys/pkg/PLAW-111publ31/pdf/PLAW-111publ31.pdf.
